# Current News

**Published:** 1900-08

**Authors:** 


### Current News.

### ILLINOIS STATE DENTAL SOCIETY.

List of officers elected for the ensuing year at the thirty-sixth annual meeting, held in Springfield, May 8 to 11, 1900:

President, J. G. Reid, Chicago; Vice-President, M. L. Hanaford, Rockford; Secretary, A. H. Peck, 92 State Street, Chicago;

Treasurer, C. N. Johnson, Chicago; Librarian, J. T. Cummins, Metropolis City; Executive Committee, C. R. Taylor, Streator; Committee on Science and Literature, A. W. Harlan, Chicago; Committee on Art and Invention, H. J. Goslee, Chicago; Supervisor of Clinics, J. E. Hinkins, Chicago.

*Members of Executive Council* (terms to expire 1903).—C. B. Sawyer, Jacksonville; M. L. Hanaford, Rockford; C. B. Rohland, Alton.

*Board of Examiners.*—C. M. Robbins, Carthage; C. C. Corbett, Edwardsville; S. F. Duncan, Joliet.

*Committee on Ethics.*—C. B. Sawyer, Jacksonville; J. D. Nicol, Peoria; Edmund Noyes, Chicago.

---

### CHICAGO DENTAL SOCIETY.

The following officers of the Chicago Dental Society for 1900-1901 were elected at the annual meeting, held in the Stewart Building, Tuesday evening, April 3, 1900:

President, Geo. W. Cook; First Vice-President, Geo. B. Perry; Second Vice-President, H. J. Goslee; Secretary, Elgin Ma Whinney; Corresponding Secretary, C. S. Bigelow; Treasurer, A. B. Clark; Librarian, H. W. Sale; Member Board of Directors, Edmund Noyes.

*Board of Censors.*—W. V.-B. Ames, Chairman; C. N. Johnson, A. W. Harlan.

C. S. Bigelow,
*Corresponding Secretary.*

---

### ODONTOGRAPHIC SOCIETY OF CHICAGO.

The following officers for 1900 were elected at the annual meeting of the Odontographic Society:

President, T. L. Gilmer; Vice-President, L. S. Tenney; Secretary, F. H. Zinn; Treasurer, G. N. West.

*Board of Directors.*—J. E. Nyman, A. B. Allen, G. B. Perry.

*Board of Censors.*—A. G. Johnson, Chairman; F. E. Roach, J. B. Dicus.

F. H. Zinn,
*Secretary.*

